# *Dunaliella salina* Microalga Restores the Metabolic Equilibrium and Ameliorates the Hepatic Inflammatory Response Induced by Zinc Oxide Nanoparticles (ZnO-NPs) in Male Zebrafish

**DOI:** 10.3390/biology11101447

**Published:** 2022-10-01

**Authors:** Suzan Attia Mawed, Gerardo Centoducati, Mayada R. Farag, Mahmoud Alagawany, Shimaa M. Abou-Zeid, Walaa M. Elhady, Mohamed T. El-Saadony, Alessandro Di Cerbo, Sheren A. Al-Zahaby

**Affiliations:** 1 Zoology Department, Faculty of Science, Zagazig University, Zagazig 44519, Egypt; 2 Department of Veterinary Medicine, University of Bari Aldo Moro, Casamassima km 3, 70010 Valenzano, Italy; 3 Forensic Medicine and Toxicology Department, Faculty of Veterinary Medicine, Zagazig University, Zagazig 44519, Egypt; 4 Poultry Department, Faculty of Agriculture, Zagazig University, Zagazig 44519, Egypt; 5 Department of Forensic Medicine and Toxicology, Faculty of Veterinary Medicine, University of Sadat City, Sadat 6012201, Egypt; 6 Department of Agricultural Microbiology, Faculty of Agriculture, Zagazig University, Zagazig 44511, Egypt; 7 School of Biosciences and Veterinary Medicine, University of Camerino, 62024 Matelica, Italy

**Keywords:** *Dunaliella salina*, zinc oxide nanoparticles, zebrafish, liver, glycogen, lipid

## Abstract

**Simple Summary:**

*Dunaliella salina*, a type of halophile green unicellular microalgae riches in carotenoids, was used to assess its ability to restore the metabolic equilibrium and mitigate the hepatic inflammation induced by zinc oxide nanoparticles (ZnO-NPs) in the male zebrafish. In our study, after 2 weeks of feeding, the body burdens of zinc residues, fish appetite, intestinal bacteria composition, and energy metabolism were restored. Additionally, the hepatic inflammation and histopathological malformations were ameliorated after *D. salina* administration.

**Abstract:**

Microalgae are rich in bioactive compounds including pigments, proteins, lipids, polyunsaturated fatty acids, carbohydrates, and vitamins. Due to their non-toxic and nutritious characteristics, these are suggested as important food for many aquatic animals. *Dunaliella salina* is a well-known microalga that accumulates valuable amounts of carotenoids. We investigated whether it could restore the metabolic equilibrium and mitigate the hepatic inflammation induced by zinc oxide nanoparticles (ZnO-NPs) using male zebrafish which were exposed to 1/5^th^ 96 h-LC_50_ for 4 weeks, followed by dietary supplementation with *D. salina* at two concentrations (15% and 30%) for 2 weeks. Collectively, ZnO-NPs affected fish appetite, whole body composition, hepatic glycogen and lipid contents, intestinal bacterial and *Aeromonas* counts, as well as hepatic tumor necrosis factor- α (TNF-α). In addition, the mRNA expression of genes related to gluconeogenesis (*pck1*, *gys2*, and *g6pc3*), lipogenesis (*srepf1*, *acaca*, *fasn*, and *cd36*), and inflammatory response (*tnf-α*, *tnf-β*, *nf-kb2*) were modulated. *D. salina* reduced the body burden of zinc residues, restored the fish appetite and normal liver architecture, and mitigated the toxic impacts of ZnO-NPs on whole-body composition, intestinal bacteria, energy metabolism, and hepatic inflammatory markers. Our results revealed that the administration of *D. salina* might be effective in neutralizing the hepatotoxic effects of ZnO-NPs in the zebrafish model.

## 1. Introduction

Nowadays, the use of zinc oxide nanoparticles (ZnO-NPs) is increasing worldwide owing to their exciting properties such as antimicrobial activity and their ability to absorb ultra violet (UV) radiation [1,2,3,4,5]. This accounts for their implication in biomedical, industrial, and customer product fields such as drug delivery, food and packaging additives, cosmetics, and personal care products [6,7]. Moreover, ZnO-NPs can be used in the agriculture field as nano-fertilizers [8], and thus enter the aquatic environment. Subsequently, the continuous generation of ZnO-NPs from various products, sewage sludge, and industrial wastes increased the concern about accumulation in aquatic ecosystems and elevated the possibility of fish dietary exposure [9,10]. 

Other previous studies declared that ZnO-NPs inhibit the hatchability, and normal growth, and induce morphological malformations and neurodevelopmental abnormalities in the zebrafish embryos [11,12] along with hematotoxicity [13], nephrotoxicity [14], reproductive toxicity [15], and immunomodulation [16] in the adult fishes. In fact, the liver is a key metabolic organ responsible for many vital functions including energy metabolism and detoxification of various environmental pollutants [17,18,19]. The hepatotoxicity associated with ZnO-NPs exposure was previously reported in the *Oreochromis niloticus* [20,21,22,23] and *Cyprinus carpio* [24] indicated by increasing the activity of aspartate aminotransferase (AST), alanine aminotransferase (ALT), and alkaline phosphatase (ALP) and the reduction in serum activity of protein and albumin all were observed along with elevation of total triglycerides and cholesterol in serum. Related to liver function, carbohydrates and lipids are the main sources of energy production in living organisms and contribute to many vital processes including development and reproduction [25]. 

Many pollutants, such as methylmercury (MeHg) and tributyltin, are known to affect the aforementioned biomolecule metabolism in mice [26] and fish liver cells (PLHC-1 and ZFL) [27], respectively. In this regard, ZnO-NPs dramatically disrupted both gluconeogenesis and glycogenolysis in the C3A hepatocarcinoma cell line [28]. Additionally, dietary ZnO-NPs affected lipid metabolism in the liver of freshwater teleost fish [29,30].

Furthermore, the toxic effects induced by ZnO-NPs are thought to be the result of Zn^2+^ released in the cytoplasm causing mitochondrial dysfunction with subsequent activation of the caspase protein-dependent apoptosis pathway [31]. Additionally, the released Zn^2+^ directly activates the endoplasmic reticulum apoptosis by modulating the expressions of stress genes and proteins without depending on the Caspase-3 mitochondrial apoptosis pathway [32]. 

Moreover, oxidative damage was demonstrated to be the main cause of ZnO-NPs-induced hepatotoxic effects including apoptosis and change in the organizational structure of the liver [33]. Briefly, the excessive production of reactive oxygen species (ROS) may cause mitochondrial damage with subsequent activation of inflammasomes and cell death via apoptosis [34,35,36]. In the same context, embryos of zebrafish exposed to ZnO-NPs exhibited transcriptional modulations of pro-inflammatory cytokines such as tumor necrosis factor-α (TNF-α) and interleukin-1β (IL-1β) [37]. In addition, ZnO-NPs upregulated the expression of IL-1β, TNF-α, and interleukin-8 (IL-8) in the intestine of *Cyprinus carpio* [38].

Therefore, an unceasing search for fish feed supplements with antioxidant and anti-inflammatory activities that can neutralize oxidative stress and subsequent inflammatory response and apoptosis of toxicants, is of primary importance [23].

Among the dietary supplements, microalgae were shown to have promising properties by improving fish growth performance and antioxidant capacity, owing to their unique content of lipids, proteins, and antioxidants [39].

*Dunaliella salina* is a microalga recently suggested as a natural feed for fish because of its high content of essential amino acids (up to 8%), protein (up to 30–50%), lipids (about 10%), carbohydrates, vitamins, and pigments [40,41]. It has been reported to be rich in valuable antioxidants including zeaxanthin [42], *β*-carotene [40], omega-3 polyunsaturated fatty acids [43], and vitamin B_12_ [44].

Furthermore, *D. salina* was reported to have antibacterial and antiviral activities [41], antioxidant and anti-inflammatory properties [35], enhance immune response [45], have a neuroprotective effect [46,47], and genoprotective activity [48]. Furthermore, *D. salina* is used in sun-protection products to protect skin from ultraviolet sun rays [40]. In addition, it exhibited a chemopreventive activity on breast cancer in rats [49] and a hepatoprotective effect against CCl_4_ [50], paracetamol [51], and thioacetamide [52].

Zebrafish (*Danio rerio*) has become a favorite model organism in ecotoxicology and drug screening research owing to its small size, high fertility and rapid organogenesis, easy breeding, and optical transparency of embryos allowing easy observation [34]. 

Moreover, the growing knowledge of the zebrafish genome, including a completed genome sequence enables toxicological findings to be increasingly related to the possible genes involved [53]. Additionally, zebrafish was reported to be a good model for the assessment of carbohydrate and lipid metabolism [54,55]. 

The present investigation aimed to evaluate the toxic impacts of ZnO-NPs and the antidotal efficacy of *D. salina* on the lipid and carbohydrate metabolism in zebrafish. To this end, hepatic glycogen and lipid contents were investigated together along with the transcription of genes related to gluconeogenesis (*pck1*, *gys1*, and *g6pc3*) and lipogenesis (*srepf1*, *acaca*, *fasn*, and *cd36*). Additionally, to assess the potential effects of ZnO-NPs on the hepatic tissue inflammatory response, TNF-α protein was measured beside the mRNA expressions of inflammatory-related genes incorporated in the nuclear factor kappa-light-chain-enhancer of activated B cells (nf-κb) pathway including *tnf*-α, *tnf*-*β,* and *nf-κb2*.

## 2. Materials and Methods

### 2.1. Zinc Oxide Nanoparticles Preparation

ZnO-NPs were prepared as follows: 12 g of Zn (NO_3_)_2_6H_2_O (Sigma-Aldrich International GmbH: St. Louis, MO, USA) were dissolved in 1 L of distilled H_2_O for a final nominal concentration of 5 mM, then stirred with sodium hydroxide (10% NaOH) solution over a hot plate magnetic stirrer for 25 min, then for 2 h at 70 °C. The obtained solution was cooled and filtrated as mentioned previously [56].

To determine the optical absorption spectra of ZnO-NPs, ultraviolet-visible spectroscopy (UV-vis) was used (LaxcoTM dual-beam spectrophotometer). In addition, transmission electron microscope (TEM) analysis was employed to determine the morphological characteristics of ZnO-NPs, particularly their size and diameter using (TEM, JEOL 1010, Japan). Dynamic light scattering analysis (DLS analysis) was utilized to measure the size of the particles in the colloidal solution (Nano Z2 Malven, Malvern Hills, UK). Finally, the zeta potential analysis was conducted to determine the surface charges of ZnO-NPs and their relative stability. 

### 2.2. Zebrafish Maintenance 

Adult male zebrafish (*Danio rerio*) were bought from a local fish supplier (Cairo, Egypt) and acclimatized for two weeks before the experiment. During the acclimatization and throughout the experiment, fishes were kept in aerated water at 27.5 ± 1 °C in glass aquaria (80 × 40 × 30 cm, water capacity 60 L) 14 h light: 10 h dark, pH 6.7 ± 0.2 and dissolved O_2_ 6.3 ± 0.5 mg per L. The fishes were fed twice a day with a commercial diet of dried bloodworm. A total of 450 fishes at 6 months of age were used in the present study (90 for determining the LC_50_ and 360 in the antidotal study).

### 2.3. Diets Formulation

Lyophilized microalgae powder of *D. salina* was kindly supplied by the National Research Center (Giza, Egypt). The strain was first cultured at 28 °C in a 500-mL flask, then the cells were harvested after 4 days and lyophilized. 

On the other side, dried bloodworms (Egymag biotechnology company, Benha, Egypt) were used as a commercial diet after grinding with a mixer into fine powder. 

To prepare a mixture of bloodworm and microalgae powder, they were mixed with an appropriate quantity of double distilled water ddH_2_O to obtain a doughy shape, which then was allowed to pass through a 0.5 mm sieve to obtain wet pellets. Then, these pellets were freeze-dried for 24 h and stored in vacuumed plastic bags at −20 °C until use. Subsequently, the fishes were fed twice a day with this commercial food throughout the experimental period (at a rate of 5% of the fish biomass). In our experiment, we tried two concentrations of *D. salina* by adding the lyophilized microalgae powder from *D. salina* to the bloodworm diet at a rate of 15% and 30% (*w*/*w*) and named D1 and D2 groups, respectively.

### 2.4. Acute Toxicity Study (Determining the Median Lethal Concentration; 96-h LC_50_)

For estimation of the LC_50_ of ZnO-NPs, acclimatized males (number = 90; weight 0.54 ± 0.13 g; length 3.2 ± 0.45 cm) were randomly divided into 9 groups, each with 10 fishes. The fishes were kept without feeding or changing the aquaria water for a continuous 4 days (96-h) and ZnO-NPs were added to the experimental group to obtain the following concentration, respectively, (0.05 mg/L, 0.1 mg/L, 0.2 mg/L, 0.4 mg/L, 0.8 mg/L, 1.6 mg/L, 3.2 mg/L, and 6.4 mg/L) besides a control group. Regularly, the outer morphology, swimming behavior, and fish mortality for all groups were recorded daily compared with the control group to calculate the LC_50_. 

### 2.5. Antidotal Study

After calculating the LC_50_, acclimatized males (n = 360) were randomly distributed into six equal groups of 60 fishes in triplicate (each has 20 fishes). The first group was used as a control. The second and third groups received *D. salina*- and dried worm-supplemented diets that were prepared previously at a rate of 15% and 30%, respectively, and named as D1 and D2 groups for 30 days. The fourth group (ZnO group) received 1/5th of the estimated LC_50_ of ZnO-NPs daily for 30 days. The fifth (ZnD1) and the sixth (ZnD2) groups received 1/5th of the estimated LC_50_ of ZnO-NPs for 30 days and then were fed on low (D1) and high (D2) *D. salina*- and dried worm supplemented diets (15% and 30%), respectively, for two weeks. Throughout the experiment, fish were kept in water at 27.5 ± 1 °C, pH 6.7 ± 0.2, and dissolved in O_2_ (6.3 ± 0.5 mg/L) and ammonia (0.045 ± 0.006 mg/L). To determine the phenotypic abnormalities of the alimentary canal, 10 fishes from each group were randomly dissected and photographed. The experimental procedures were carried out at the Zoology Department, Faculty of Science, Zagazig University, Zagazig, Egypt.

### 2.6. Determination of Fishes’ Whole Body Chemical Composition 

By the end of the experiment, 5 fishes were randomly selected from each replicate in all experimental groups and used to estimate the chemical composition of the fishes’ whole body (%) on a wet weight basis [57]. The content of crude protein was measured by the Kjeldahl Distillation Unit (Velp Scientifica, Via Stazione, Italy) [58]. Natural convection oven (JSON-100, Republic of Korea) was used to determine the moisture percentage. Moreover, the ash constituents and crude lipids were determined by Muffle Furnaces (Thermo Scientific, Waltham, MA, USA) and Soxhlet extractor glassware, respectively.

### 2.7. ZnO-NPs Residues Assessments in the Whole Fish Body

Homogenates of 5 fishes per each experimental group were exposed to acid digestion. After that, 1 g of each sample was digested in a screw-capped glass bottle by adding 4 mL of digestion solution consisting of perchloric acid/nitric, 1:1 *v*/*v* [59]. Initial digestion was conducted at room temperature and lasted for 24 h followed by heating for 2 h at 110 ˚C. Then, the mixture was allowed to cool followed by adding deionized water, and then the obtained solution was warmed for 1 h in a water bath to expel nitrous gases. Digests were then filtered using Whatman No. 1 and diluted to 25 mL of deionized water [60]. 

The obtained resultant solution was then analyzed by using the flame atomic absorption spectrophotometer (FAAS).

### 2.8. Determination of Total Intestinal Bacteria and Aeromonas Counts

Randomly, fish intestine samples were taken from each group to compute the total bacterial and *Aeromonas* counts (n = 5). The samples were homogenized in a sterilized screw bottle with sterile saline peptone water (8.5 g/L NaCl and 1 g/L peptone). Then, this solution was serially diluted up to 10^7^. The bacterial count in fish samples was represented as log CFU g-1. The total bacterial count was determined on a plate count agar (PCA) at 37 °C for a day [61], whereas *Aeromonas* counts were determined on *Aeromonas* agar medium after a day of incubation at 37 °C [62]. 

### 2.9. Assessments of Hepatic Glycogen, Lipids, and Histopathological Analysis

Dissected livers from the experimental groups were fixed in 10% *v*/*v* neutral formalin for 1 week at 4 °C and then processed sequentially in ethanol, xylene, and paraffin wax. 

Tissue sections of 5 μm thickness were prepared using a microtome (Leica Model, RM2125 Biosystems, Deer Park, NY, USA). The sections were subsequently stained with hematoxylin and eosin for histopathological detection, periodic acid-Schiff (PAS) for liver glycogen content, and Oil Red O (ORO) for lipid detection. The scanning and analysis method was performed by the PANNORAMIC MIDI I (Digital Slide Scanners MIDI, 3D HISTECH Company, Budapest, Hungary). After the tissue slice is put on the machine, the slice gradually moves under the lens of the scanner, while moving and imaging, all the tissue information on the slice is captured. All are scanned and imaged to form a file that contains all the tissue information on the tissue section. After the file is opened with panoramic viewer software, it can be magnified at any multiple of 1–400 times for observation and pictures can be captured at any part data were put in an excel sheet and graphs were obtained with graph pad prism 8 (GraphPad Software, La Jolla, CA, USA) using the mean of 3 readings for each area. For glycogen, the purple area was the main goal to identify the density for the purple color. On the ORO, the red color is the main goal so the red density was identified is illustrated as numbers also in the excel sheet. [63]. 

### 2.10. RNA Extraction and Real-Time Quantitative Polymerase Chain Reaction (qRT-PCR)

Total RNA was extracted from homogenates of 5 liver tissues from each experimental group using TRIzol reagents according to the manufacturer’s instructions (Life Technologies, Carlsbad, CA, USA). RNA purity and integrity were evaluated by gel electrophoresis and the ratio of absorbance at 260/280 nm of UV5 Nano Microvolume spectrophotometer (METTLER, Toledo, ON, Canada), respectively. A total of 1 μg of the total extracted RNA was reverse transcribed into cDNA by PrimeScript TM RT reagent Kit with gDNA Eraser (Stratagene, Japan, Takara). Quantitative real-time PCR was performed on the MSLPCR30 Thermal Cycler system (Biobase Biozone Co., Ltd., Guangdong, China), and the housekeeping gene β-actin was used as an internal control to normalize the expression values. 

The gene-specific primers were designed with the primer premier 5.0 software (BIOPROCESS ONLINE, Pitssburg, CA, USA) (Table 1) and the amplification protocol of qRT-PCR was previously described [64].

### 2.11. Immunohistochemistry and Image Analysis

Paraffin sections were dewaxed and rehydrated. Slides emerged in sodium citrate buffer (pH 6.0) for 20 min at 95 °C for antigen retrieval and then treated with 0.3% H_2_O_2_ for 10 min to inactivate the endogenous enzyme. To reduce unspecific binding, sections were incubated in phosphate-buffered saline (PBS) (0.02 M, PH 7.4) containing 5% Bovine Serum Albumin (BSA) for 1 h at 37 °C. Thereafter, sections were incubated with the first antibody Anti-TNF-α antibody 52B83 (1:50 dilution, Abcam 52B83, ab1793, Cambridge, UK) at 4 °C overnight. 

After 3 washes with PBST, the sections were incubated with 100 µL of the secondary antibody (Horse Radish Peroxidase HRP) for 1 h at room temperature. Finally, the specificity of the reactions obtained was colored by using DAB (3, 3′-diaminobenzidine substrate) and counterstained with hematoxylin for nuclear differentiation. Histological images were captured by Case Viewer software. Images quantification and analysis were performed by 3D Histech Quant Center using a tissue slice scanner model (Pannoramic MIDI, 3DHISTECH Kft., Budapest, Hungary) and the quantitative analysis was performed by panoramic viewer software. The magnification power covers different areas (10 X = 100 µm, 100 X = 10 µm, 40 X = 25 µm, 400 X = 2.5 µm), the brown color of the area is the main goal so the immunohistochemical scoring of TNF-α (brown positive expression) was calculated between the groups [65]. 

### 2.12. Statistical Analysis 

All data were analyzed using SPSS 17.0 (SPSS, Inc, IBM^®^, New York, NY, USA). The statistical analysis was performed using a two-tailed Student’s *t*-test when comparing two groups and one-way ANOVA for comparison of more than two groups. In all analyses, the *p* value was presented as the following; (* *p* < 0.05; ** *p* < 0.01; *** *p* < 0.001). Graphics and plots were designed using GraphPad Prism 8 software package (GraphPad Software, La Jolla, CA, USA) [66].

## 3. Results

### 3.1. ZnO-NPs Characterization 

**Figure 1:**
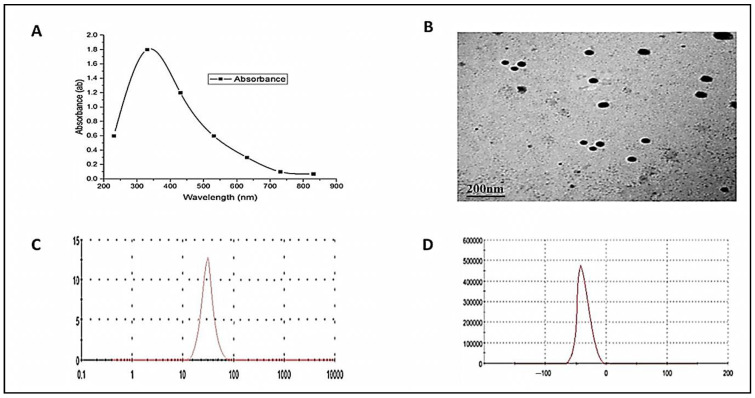
Characterization of ZnO-NPs by four devices: UV–VIS spectroscopy analysis showed the maximum peak at 340 nm (**A**); the ZnO-NPs were spherical with an average size of 108 nm (**B**); the size was estimated based on the Brownian motion of the ZnO-NPs in suspension, where the exact size was 89 nm (**C**); the zeta potential analysis was carried out to determine the surface charge of ZnO-NPs, which ensures the stability of synthesized nanoparticles, where the net surface charge was −33 mV (**D**).

Figure 1 shows the characterization of ZnO-NPs by four devices. The results showed a maximum peak at 340 nm (Figure 1A). Additionally, ZnO-NPs appeared spherical with an average size of 108 nm (Figure 1B). On the other side, the size was estimated based on the Brownian motion of the ZnO-NPs in suspension, where the exact size was 89 nm (Figure 1C). Finally, the zeta potential analysis was carried out to determine the surface charge of ZnO-NPs, which ensures the stability of synthesized nanoparticles where the net surface charge was −33 mV (Figure 1D).

### 3.2. The Value of Estimated 96-h LC_50_ and Behavioral Responses in ZnO-NPs Exposed Male Zebrafish 

There were no mortalities or abnormal behaviors in the control group during the study period (96 h). Contrarily, the ZnO-NPs exposed fishes showed dose-dependent mortalities. The 96-h LC_50_ of ZnO-NPs was estimated to be 3.48 mg/L with lower and upper confidence limits of 2.49 and 5.37 mg/L, respectively. There were varying degrees of behavioral changes observed both in live and dead fishes, in a dose-dependent way. Fishes exhibited abnormal swimming (uncoordinated), speed and sluggish motion, and surface mouth breathing. Stressed fishes showed tail sinking, increasing mouth breathing, and hyperventilation (Table 2). 

### 3.3. D. salina Restores the Appetite Loss of ZnO-NPs Exposed Males 

**Figure 2:**
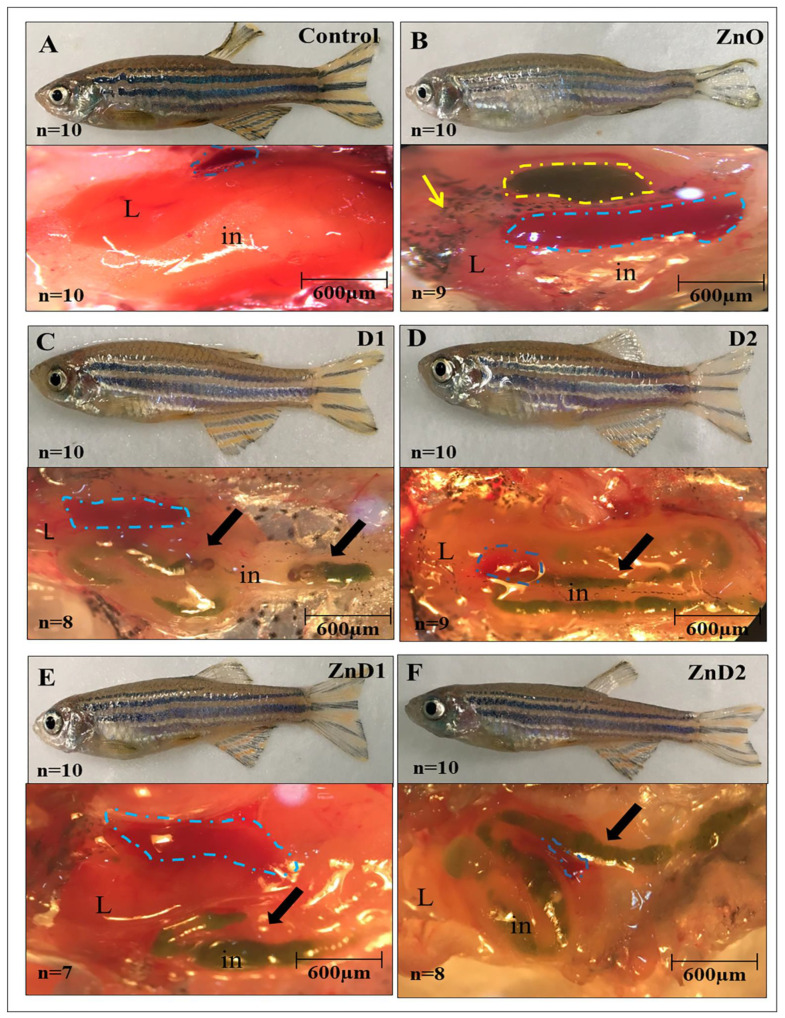
*D. salina* restored the fish appetite after ZnO-NPs exposure: (**A**) Representative pictures of the control zebrafish with a normal phenotype of the liver, intestine, and spleen (Blue dashed line); (**B**) photographs for ZnO-NPs exposed fish with whitish skin color, feeble irregular body, swollen gallbladder (Yellow dash line), enlarged spleen (Blue dash line), and abnormal liver (Yellow arrow); (**C**) photographs of the D1 group with normal shape and liver morphology; (**D**) photographs of the D2 group with normal morphology; notice the presence of the green microalgae inside the fish intestine (Black arrow); (**E**) photographs of ZnD1 group; the dissected intestine contains green *D. salina* (Black arrow) despite the abnormal body shape and skin coloration; (**F**) photographs of the ZnD2 group exhibiting normal body shape; and the liver, spleen, and intestine appeared to have more green algae (Black arrow). The number of dissected males has the same phenotype that is shown in the pictures (n/10). L: liver, in: intestine.

Control fishes, as well as D1 and D2 groups, showed normal body morphology and swimming motion. On the other hand, fishes of the ZnO-NPs exposed group lost their appetite after 4 days post-treatment (4 dpt) with a significant reduction in body weight and abnormal skin color at the end of the experiment. On the dissection, control fishes showed normal livers and intestines full of food (Figure 2A). Contrarily, ZnO-NPs exposed males manifested feeble bodies (weight, 0.25 ± 0.11 g) compared with the control group (0.54 ± 0.13 g), swollen gallbladder harboring a large accumulation of green bile salts, and significant spleen enlargement. Interestingly, the intestine of the dissected ZnO-NPs males showed crude intestinal secretions without food materials inside (Figure 2B). 

Males from D1 and D2 groups presented normal outer morphology with normal liver, spleen, and intestine packed with green *D. salina* (Figure 2C,D). To investigate the metabolic function of *D. salina*, ZnO-NPs treated males were fed on *D. salina* (low and high levels) for extra 2 weeks. Herein, fishes retrieved their appetite, body shape, and weight gain (0.35 ± 0.12 g), and began to swim slowly. Collectively, *D. salina* restored the normal gallbladder and spleen morphology after ZnO-NPs exposure. Moreover, the intestine appeared filled with *D. salina,* particularly in the ZnD2 group demonstrating improvement in food intake (Figure 2E,F).

### 3.4. Determination of ZnO-NPs Residues and their Effects on the Whole-Body Composition 

Analysis of ZnO-NPs residues in the whole body of the treated fishes is represented in Table 3. 

The results revealed that the highest concentration of accumulated ZnO-NPs was detected in the body of fishes exposed to ZnO-NPs for 4 weeks. Post-exposure to *D. salina* after ZnO-NPs caused a significant decrease in the residual accumulation of ZnO-NPs in the tissue of fishes, and this reduction was more obvious in the higher *D. salina* fed group (ZnD2). 

On the other side, a significant variation in the whole-body composition was not observed between the control and treated fish groups, except for the lipid content, which was lower in the ZnO-NPs exposed fishes compared with the control and other groups (Table 4).

### 3.5. D. salina Reduced Intestinal Total Bacterial and Aeromonas Counts

**Figure 3:**
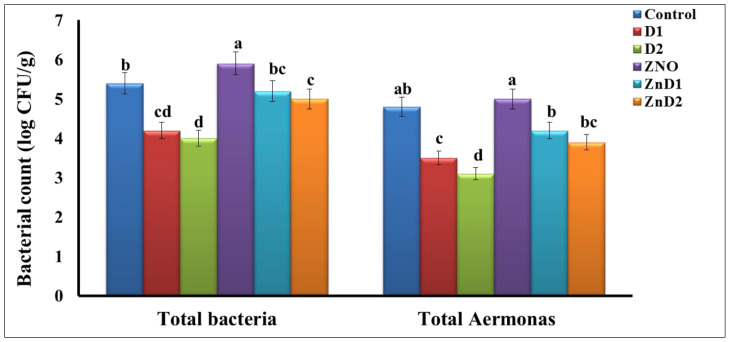
Total bacterial and *Aeromonas* count in the fishes’ intestines. Values among groups are not sharing a common superscript letter (a, b, c, d) differ significantly at *p* < 0.05.

Figure 3 shows the total bacterial and *Aeromonas* counts in the fishes’ intestinal samples. Here, the addition of *D. salina* particularly D2 showed the highest reduction level of the total bacterial and *Aeromonas* counts compared with the control males. On the other hand, the bacterial and *Aeromonas* species counts were higher in ZnO group compared with other experimental groups. 

Exposure of the ZnO group to D1 and D2 for two weeks showed a reduction in the *Aeromonas* and total bacterial counts compared with the ZnO group. 

### 3.6. D. salina Ameliorates the Hepatic Histopathological Changes Induced by ZnO-NPs 

**Figure 4:**
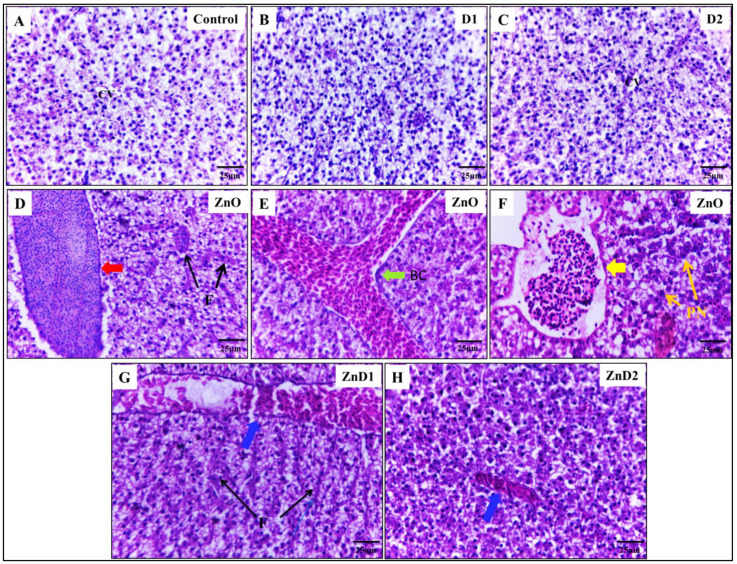
ZnO-NPs induced hepatic histopathological changes in the male Zebrafish: (**A**) Representative photomicrographs showing normal histology of hepatocytes and central vein; (**B**) photomicrograph of D1 group liver showing normal hepatic architecture; (**C**) photomicrographs of D2 group liver showing normal hepatocytes and central vein; (**D**–**F**) representative photomicrographs of the ZnO-NPs group showing different histopathological observations including inflammation (Red arrow), fibrosis (Black arrow), hemorrhagic appearance with dilation of hepatic bile ducts (Green arrow), Pyknosis (Orange arrow), and Necrosis (Yellow arrow); (**G**) representative photomicrograph of ZnD1 liver showing gradual disappearance of hemorrhagic appearance (Blue arrow) despite the presence of fibrosis (Black arrow); (**H**) representative photomicrograph of ZnD2 liver showing the ameliorative effect of *D. salina* in restoring the liver architecture, relieving hepatic inflammation and hemorrhagic appearance (Blue arrow). Scale magnification is shown in pictures. CV: Central Vein, F: Fibrosis, PN: Pyknotic Nuclei.

Histological analysis for the control, D1 and D2 groups exhibited normal hepatic architecture with defined hepatocytes and central veins (Figure 4A–C). On the other hand, the ZnO-NPs group showed significant histopathological alterations in the liver due to inflammatory cellular infiltrations. Accordingly, inflammatory infiltration activates hepatic stellate cells, the main source of the myofibroblast in the liver, resulting in increased hepatic fibrosis [67] (Figure 4D). Additionally, ZnO-NPs induced hepatic hemorrhagic appearance with dilation of the bile ducts that appeared packed with numerous blood cells (Figure 4E). Furthermore, ZnO-NPs could potentially cause adverse effects on the hepatocyte nuclei leading to hepatocyte vacuolation and karyopyknosis causing cellular necrosis (Figure 4F). Interestingly, ZnD1 and ZnD2 males began to restore their normal hepatic histology, with a significant reduction in hepatic allergy, fibrosis, and hemorrhagic appearance after feeding on *D. salina*, especially in the ZnD2 group (Figure 4G,H).

### 3.7. ZnO-NPs Altered Hepatic Glyco-Lipid Equilibrium 

**Figure 5:**
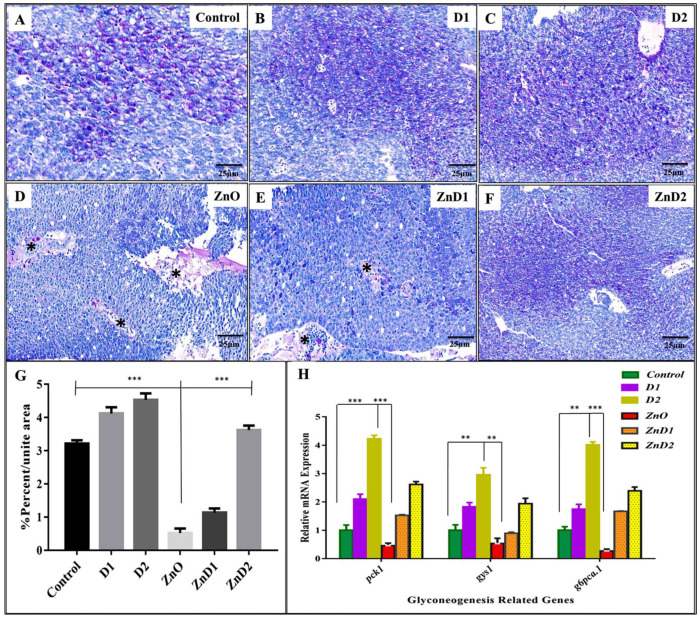
*D. salina* restored fish appetite and induced hepatic gluconeogenesis: (**A**–**C**) Periodic acid Schiff (PAS) stained liver sections of control zebrafish, D1, and D2 groups showing normal glycogen contents; notice glycogen content in the D2 group is more than in D1 and the control groups (purple staining); (**D**) representative photomicrograph of ZnO-NPs exposed group stained with PAS showing hepatic tissue degeneration, inflammatory cellular infiltration (Black asterisk), and absence of hepatic glycogen; (**E**,**F**) representative photomicrographs of PAS staining for ZnD1 and ZnD2 groups, respectively. ZnD2 is more effective in restoring liver structure and hepatic glycogen content; (**G**) statistical analysis of the glycogen-covered area (the average percentage for triple readings); (**H**) mRNA expression evaluated by qRT-PCR for gluconeogenesis-related genes indicating downregulation of gene expression in the ZnO-NPs group, which is upregulated after dietary supplementation with *D. salina*, especially in ZnD2 groups. Magnification is shown in all pictures. The results are shown as the mean ± SD.* *p* < 0.05 (significant), ** *p* < 0.01(highly significant), *** *p* < 0.001 (very high significant).

To detect hepatic metabolic equilibrium, liver glycogen was stained with Periodic Acid-Schiff (PAS). Herein, hepatocytes of the control, D1, and D2 groups had adequate amounts of glycogen that increased in the D2 group (Figure 5A–C). Contrarily, the ZnO-NPs group revealed severe hepatic tissue degeneration and inflammatory cellular infiltration with a significant reduction in the glycogen content (Figure 5D). On the other side, the ZnD1 and ZnD2 groups showed gradual aggregations of glycogen content that became more prominent in the ZnD2 group than in ZnD1 (Figure 5E,F). Here, ZnO-NPs males showed a statistically significant hepatic glycogen reduction than the control group, which could be restored via *D. salina* intake (Figure 5G). To ensure our results, mRNA expression of gluconeogenesis-related genes including *pck1*, *gys1*, and *g6pc3* genes was down-regulated in the ZnO-NPs group compared with the control, D1, and D2 groups indicating the adverse effect of ZnO-NPs on the glycogen content that could be retrieved again after *D. salina* intake in ZnD1 and ZnD2 (Figure 5H).

**Figure 6:**
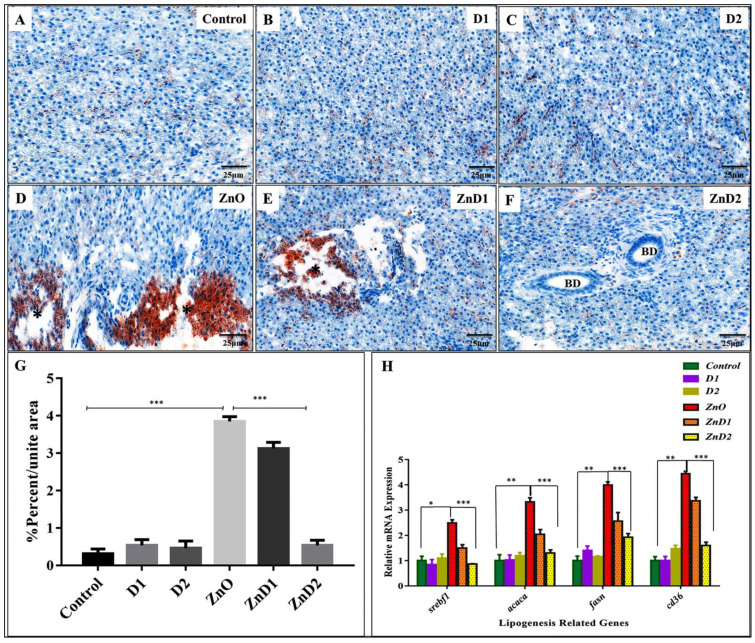
*D. salina* reduced hepatic steatosis induced by ZnO-NPs: (**A**–**C**) ORO staining of the liver in Control, D1, and D2 groups showing normal hepatocytes and a significant lipid reduction; (**D**) ORO staining of ZnO-NPs exposed group liver showing tissue degeneration (Black strikes) with lipid accumulation (Red staining); (**E**) ORO staining of ZnD1 liver showing a transition state of lipid reduction; (**F**) ORO staining of ZnD2 liver exhibiting complete recovery with significant dilation of bile ducts; (**G**) statistical analysis of ORO photomicrographs presenting the induced action of ZnO-NPs in lipid elevation and the therapeutic effect of *D. salina*; (**H**) expressions of mRNA were evaluated by qRT-PCR for lipogenesis-related genes revealing overexpression in the ZnO-NPs group compared with their siblings from the control, D1, and D2 males. In the ZnD2 group, the expression of genes was downregulated. Scale magnifications are shown in pictures and data expressed as mean ± SD. * *p* < 0.05 (significant), ** *p* < 0.01 (highly significant), *** *p* < 0.001 (very high significant).

At the same time, lipid content was stained with Oil Red O (ORO). ORO showed remarkable hepatic degeneration with remarkable lipid steatosis in the ZnO-NPs group (Figure 6D) compared with control, D1, and D2 groups that showed normal hepatic architecture without lipids retention (Figure 6A–C). In the ZnD2 group (Figure 6F), the hepatic tissue restored normal organization except for some dilation in the bile ducts without lipid contents, compared with ZnO-NPs (Figure 6D) and ZnD1 groups (Figure 6E). 

Statistical analysis of ORO photomicrographs (Figure 6G) showed the activity of ZnO-NPs in inducing steatosis and the therapeutic effect of *D. salina*. At the genetic level, mRNA expression of genes involved in lipogenesis including *srepf1, acaca,* and *fasn* was up-regulated in the ZnO-NPs group compared with their siblings from the control, D1, and D2 males. Furthermore, *cd36* (fatty acid translocase) accounts for long-chain fatty acids uptake by the hepatocytes [68] and was highly induced in the ZnO-NPs group. 

Interestingly, mRNA expression of these genes was down-regulated again in the ZnD2 group (Figure 6H). 

### 3.8. Hepatic Inflammation Could Be Effectively Prevented by D. salina after ZnO-NPs Exposure

TNF-α is an important pro-inflammatory cytokine that mediates liver inflammation, fibrosis, and hepatocyte necroptosis [69]. Here, immunohistochemistry analysis was performed to investigate the expression level of TNF-α protein in the hepatic tissue. 

**Figure 7:**
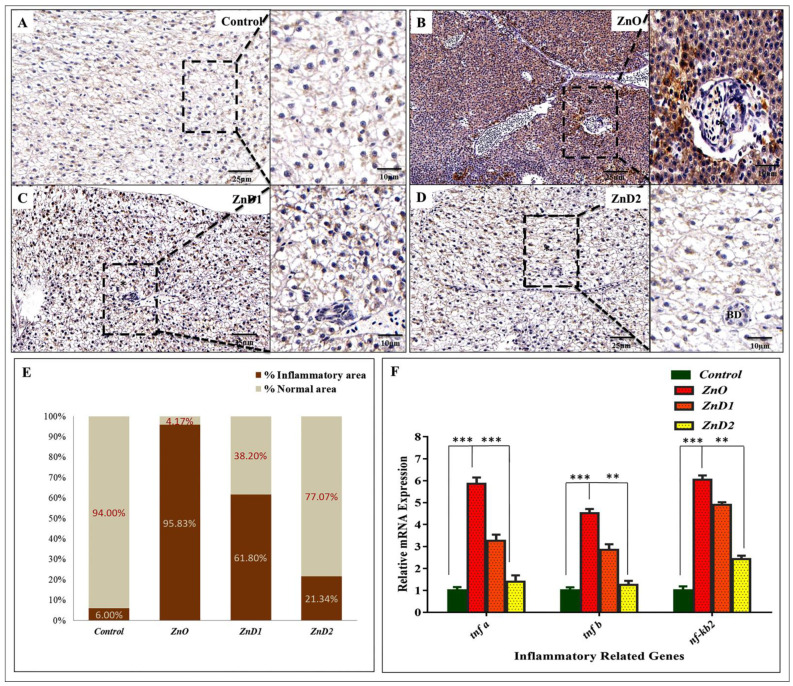
The ameliorative effect of *D. salina* on the liver inflammation induced by ZnO-NPs: (**A**,**B**) Representative photomicrographs of TNF-α immunohistochemistry of liver sections in the control and ZnO-NPs groups showing no positive reaction for TNF-α in the hepatocytes of the control group compared with the ZnO-NPs group; (**C**,**D**) representative photomicrographs of ZnD1, and ZnD2 groups showing weak expression of TNF-α (The brown color indicating TNF-α positively); (**E**) the mean cell number per unit area was quantified by NIH Image J. The results were normalized from three independent sections of the same area; (**F**) mRNA expression of genes involved in the (NF-κB) inflammatory signaling pathway. Scale magnifications were shown in pictures and data expressed as means ± SD. * *p* < 0.05 (significant), ** *p* < 0.01 (highly significant), *** *p* < 0.001 (very high significant).

ZnO-NPs exposed group showed a significant increase in TNF-α protein (Figure 7B) compared to the control group, which showed negatively staining with TNF-α (Figure 7A). The groups fed on *D. salina* after ZnO-NPs exposure also exhibited a remarkable TNF-α protein reduction compared with the ZnO-NPs group (Figure 7C,D). Furthermore, in the ZnO-NPs group, the hepatic tissue manifested advanced growth of necrosis with inflammatory cellular infiltration where the inflammatory area density covered more than 95% of the liver tissues compared with other groups (Figure 7B,E). Additionally, mRNA expression of inflammatory-related genes incorporated in the NF-kB pathway including *tnf-β, tnf-α, and nf-κb2* was significantly up-regulated in ZnO-NPs exposed group more than in the control, ZnD1, ZnD2 groups indicating the anti-inflammatory effect of *D. salina* and its role in restoring liver structure and function (Figure 7F).

## 4. Discussion

Zebrafish (*Danio rerio*) has emerged as an exciting model organism for toxicology studies due to its genetic and physiological homology with mammals and similarities in carbohydrate and lipid metabolism [70,71]. In addition, oxidative stress and inflammation parameters are the most common categories of assays applied in the zebrafish ecotoxicological investigations [72]. The present study was conducted to explore the antidotal efficacy of *D. salina* on the glycolipid metabolic disturbance and hepatic inflammation induced by ZnO-NPs in zebrafish [56]. In accordance with our findings, Jacob et al. reported that the ZnO-NPs synthesized by *A. niger* were spherical with a diameter range of 39.4–82.6 nm [73]. Herein, we demonstrated that the UV spectrum of ZnO-NPs reached its maximum peak at 340 nm. Similarly, the UV spectrum of ZnO-NPs was between 320 and 390 nm [74] and peaked at 375 nm [75].

Fishes were reported to absorb and accumulate metals in various tissues from the surrounding environment [76] and ZnONPs might be one of those particles. Our findings demonstrated that the 96-h LC_50_ of ZnO-NPS was estimated to be 3.48 mg/L in zebrafish. 

The value of LC_50_ is in the average between some previously determined values, which declared that the 96-h LC_50_ of ZnO-NPs in adult zebrafish was 3.97 mg/L [77] and 4.92 mg/L [78]. However, the differences in such values resulted from the differences in the age, rearing conditions, and chemical composition of water and food. In the present study, varying degrees of behavioral alterations were recorded in ZnO-exposed living and dying zebrafish (before death) in a dose-dependent manner. In agreement with our findings, ZnO-NPs were reported to affect locomotor activity and behavior, and to express neurodevelopmental abnormalities in zebrafish larvae [12,79]. The behavioral alterations were suggested to be attributed to dopamine neuronal loss and apoptosis in the brain of zebrafish [12]. Generally, nanoparticles are known to induce neurodegeneration in the brain tissue through oxidative stress induction, protein aggregation, apoptosis, and inflammation [80] and similar effects were obtained in the present study, however in different organs. 

Moreover, in our study, exposure to ZnO-NPs resulted in a loss of appetite and a reduction in body weight. Herein, the whole-body composition revealed a reduction in the lipid content compared with the control and other groups. Similar to our findings, Rashidian et al. recorded reduced growth performance in *Cyprinus carpio* exposed to ZnO-NPs [38]. Furthermore, ZnO-NPs were reported to inhibit zebrafish’s normal development and growth affecting the expression of cell cycle-related genes [81].

On the other side, ZnO-NPs increased intestinal bacterial and *Aeromonas* counts. This could be returned to the antibacterial activity of ZnO-NPs on intestinal bacteria, probably through the generation of ROS and subsequent bacterial cell disruptions [82].

Furthermore, feeding on *D. salina* for 2 weeks after ZnO-NPs exposure retrieved fish appetite, body shape, weight, normal gallbladder and spleen, and fishes began to swim slowly. In agreement with our results, *D. salina* has been shown to increase growth in zebrafish [83], *Oncorhynchus mykiss,* and *Oreochromis* species [41].

The increase in body weight could be produced by the variety of nutritional elements in *D. salina* including protein, carbohydrates, and lipids. In addition, the high *β*-carotene content was demonstrated to increase *Oreochromis niloticus* growth [84].

Therefore, the improvement of growth performance could also be attributed to modulation of the immune status and gut beneficial microbiota [83]. This agrees with Pratiwi, who reported that *D. salina* improved the performance of *Oreochromis niloticus*, probably through its ß-carotene content which can increase the fish’s antioxidant capacity [41].

At the histopathological level, ZnO-NPs in this study induced hepatic tissue degeneration and inflammatory cellular infiltration, Liu et al. observed that zebrafish exposed to ZnO-NPs showed edema, cytoplastic vacuolation, and pyknotic nucleus [33]. The number of hepatic macrophages deposited, and the sinus clearance was increased. These effects were associated with increased malondialdehyde (MDA) hepatic content and suppression of antioxidant enzymes with upregulation of apoptosis-related genes. 

Moreover, Rajkumar et al. demonstrated that administration of ZnO-NPs to *Cyprinus carpio* resulted in degeneration of liver tissues, vacuolization, cell infiltration, and nuclear alteration [13]. Furthermore, ZnO-NPs were reported to damage the hepatic tissue as evidenced by elevated liver enzyme markers in the serum of *Oreochromis niloticus* and *C. carpio* [20,21,24] and decreased serum albumin and total protein [21]. The induced structural changes in the liver of ZnO-NPs-exposed fishes could be attributed to hepatic oxidative damage with an increased rate of lipid peroxidation and suppression of antioxidant enzymes [11,33].

Notably, *D. salina* has hepatoprotective effects in the form of improvement of liver histopathology and serum biomarkers that were previously reported in rats intoxicated with CCl_4_ [50], paracetamol [51], and thioacetamide [42,52]. This effect was suggested to be mediated by the antioxidant and anti-inflammatory capacity of *D. salina* owing to its high total carotenoid content, especially β-carotene in addition to the unsaturated fatty acids such as alpha-linolenic acid [42].

In the current investigation, the ZnO-NPs group manifested a significant reduction in the glycogen content at the histological and genetic levels. It was also in agreement with Filippi et al. who reported that the hepatocarcinoma cells exposed *in vitro* to ZnO-NPs presented a dose-dependent increase in glycogen breakdown, with an elevation of glucose release and glycolysis. Hence, the reduction in the hepatic glycogen content could be attributed to increased glycogen breakdown and downregulation of the expression of gluconeogenesis-related genes [28].

On the other side, lipid metabolism is the main key to energy production that controls different physiological, reproductive, and developmental processes [55]. In our study, remarkable hepatic degeneration with lipid steatosis was observed in the ZnO-NPs-exposed group at the histological and genetic levels. It agrees with the previous findings demonstrated by Chen et al. who declared that ZnO-NPs disrupted the lipid metabolism in the intestine of freshwater teleost yellow catfish *Pelteobagrus fulvidraco* [29]. 

Furthermore, it increased the triglyceride contents and the activity of the lipogenic enzymes besides the upregulation of the lipogenic genes including *6pgd*, *fas*, and *srebp1*, and the downregulation of small heterodimer partner (*shp*) and farnesoid X receptor (*fxr*). In addition, He et al. recorded hepatic steatosis in ZnONPs-treated zebrafish [11]. Collectively, this could result from the promotion of fatty acid synthesis via activation of the *srebp* gene and its downstream genes *fasn* and *acc1*. The author attributed this to the generation of ROS with subsequent endoplasmic reticulum stress (ERS). The ERS activates the *srebps*, which are the most important transcription factors present in the endoplasmic reticulum [85]. They are involved in the regulation of lipogenesis-related genes such as *srebp1c* leading to liver steatosis. In the current work, we recorded the upregulation of expression of hepatic *srebf1* (a zebrafish homolog of *srebp1c*), which can activate enzymes involved in lipogenesis and fatty acid synthase (*fasn*), *acaca*, and stearoyl-CoA desaturase (*scd*) [86,87,88].

The effects of *D. salina* on the metabolic equilibrium after ZnO-NPs exposure agrees with those of El-Baz, who demonstrated that *D. salina* ameliorated the D-galactose-induced hepatic steatosis in rats via mitigation of oxidative damage, and apoptotic and inflammatory indices [89]. This action was demonstrated to be mediated through the carotenoid fraction and zeaxanthin present in *D. salina*. β-carotene is a powerful antioxidant that can counteract the ROS possibly through modulation of the Nrf2/ARE pathway [90]. *β*-carotene is a vitamin A precursor, and therefore directly affects cholesterol synthesis [91]. The carotenoid is metabolized to retinoic acid, which regulates the expression of genes responsible for many metabolic processes [92].

In our study, ZnO-NPs-exposed fish exhibited a significant increase in TNF-α protein. The hepatic tissue manifested advanced growth of necrosis with inflammatory cell infiltration at the histological and genetic levels. In agreement with our findings, Brun et al. reported that ZnO-NPs produced transcriptional changes in pro-inflammatory cytokines TNF-α and IL-1β in zebrafish embryos [37]. Similarly, Rashidian et al. demonstrated that ZnO-NPs upregulated Il-1β, TNF-α, and IL-8 in *Cyprinus carpio* exposed to ZnO-NPs [38]. Additionally, Tan et al. reported that the ultrafine particulate matter can activate Kupffer cells in animal models and thus exacerbate non-alcoholic fatty liver disease (NAFLD) [93]. Feeding on *D. salina* after ZnO-NPs exposure exhibited a remarkable TNF-α protein reduction and downregulation of inflammatory genes incorporated in the NF-kB pathway indicating the ameliorative effect of *D. salina* in restoring liver structure and function. Similarly, the anti-inflammatory potency of *D. salina* was previously reported in zebrafish at the normal physiological state, where the expressions of intestinal IL-6, IL-8, and IL-1β were decreased [83]. A similar impact was recorded by Abdel-Daim et al. against ulcerative colitis induced by acetic acid in rats where colon myeloperoxidase (MPO), prostaglandin 2 (PGE2), TNF-α, IL-6, and IL-1β showed improvement upon *D. salina* treatment [94]. Lin et al. mentioned that *D. salina* suppressed IL-6, nitric oxide (NO), and ROS production and downregulated cyclooxygenase-2 (COX-2) and inducible nitric oxide synthase (iNOS) expression in virus-infected murine macrophage cells [95]. 

This anti-inflammatory activity was attributed to the regulation of NF-κB expression. A similar anti-inflammatory effect was produced by *D. salina* in rats with intestinal injury produced by gamma irradiation [96]. Moreover, *D. salina* showed anti-inflammatory effects in rats with diabetic neuropathy induced by streptozotocin [46] and thioacetamide-induced hepatic encephalopathy [52].

Furthermore, the anti-inflammatory action of *D. salina* may be related to its contents of Omega-3 fatty acids and zeaxanthin. The *D. salina*-Omega-3 fatty acid was found to block NF-κB nuclear translocation and downregulate the inflammatory markers produced by peripheral blood mononuclear cells [43]. The *D. salina*-zeaxanthin was demonstrated to mitigate the D-galactose-induced aging dementia in rats by decreasing the brain level of IL-1β and iNOs [97,98].

## 5. Conclusions

Our results demonstrated that ZnO-NPs are hepatotoxic in zebrafish, affecting fish appetite and whole-body composition by disrupting carbohydrate and lipid metabolism. 

ZnO-NPs altered the intestinal bacterial and *Aeromonas* counts, and hepatic TNF-α and modulated the expression of the gluconeogenesis, lipogenesis, and inflammatory-related genes. Depending on the biochemical analysis, histopathology, and gene expression studies, we have demonstrated the antidotal activity effect of *D.* *salina* against hepatotoxic and metabolic disorders in ZnO-NP-exposed fish. The underlying mechanisms included restoring the glycolipid equilibrium and anti-inflammatory activity through modulation of the NF-κB. Thus, *D. salina* is suggested as a valuable feed additive for fish. 

## Figures and Tables

**Table 1:** Primer sequences (forward and reverse) used for real-time qPCR analysis.

Gene	Accession (Gene ID)	Sequences (5′–3′)	Gene Name
Gluconeogenesis			
*pck1*	NM_214751	F: 5′ ATCACGCATCGCTAAAGAGG 3′	Phosphoenolpyruvate carboxykinase 1
		R: 5′ CCGCTGCGAAATACTTCTTC 3′	
*gys1*	NM_201180	F: 5′ GCAGCTCAGTGTGACGAACC 3′	glycogen synthase 1
		R: 5′ GGTCCCCTGCTTCCTTATCC 3′	
*g6pca.1*	NM_001003512	F: 5′ TCACAGCGTTGCTTTCAATC 3′	glucose-6-phosphatase a, catalytic subunit, tandem duplicate 1
		R: 5′ AACCCAGAAACATCCACAGC 3′	
Lipogenesis			
*srepf1*	NM_001105129	F: 5′CATCCACATGGCTCTGAGTG 3′	sterol regulatory element binding transcription factor 1
		R: 5′CTCATCCACAAAGAAGCGGT 3′	
*acaca*	NM_001271308	F: 5′GGACGGACCCTTGCACAATA 3′	acetyl-CoA carboxylase 1
		R: 5′CCTCTGCAGGTCGATACGTC 3′	
*fasn*	XM_009306807	F: 5′GAGAAAGCTTGCCAAACAGG 3′	Fatty acid synthase
		R: 5′GAGGGTCTTGCAGGAGACAG-3′	
*cd36*	NM_001002363	F: 5′AGGCCACTGTGAACCTGAAG 3′	Thrombospondin receptor
		R: 5′AAGTTGGGGTTCATTCCGAC 3′	
Inflammation			
*tnf-α*	NM_212859	F: 5′AGACCTTAGACTGGAGAGATGAC 3′	Tumor necrosis factor α
		R: 5′ CAAAGACACCTGGCTGTAGAC 3′	
*tnf-β*	NM_001024447	F: 5′TCAGAAACCCAACAGAGAACATC 3′	tumor necrosis factor β
		R: 5′ ACCCATTTCAGCGATTGTCC 3′	
*nf-κb2*	NM_001001840	F: 5′ATGAGAACGGAGACACG 3′	kappaB kinase/NF-kappaB cascade
		R: 5′CAGCAATCGCAAACAA 3′	
β-actin			
*β-actin*	NM_131031	F: 5′ATGGATGAGGAAATCGCTGC 3′	actin, beta 1 (actb1)
		R: 5′CTTTCTGTCCCATGCCAACC 3′	

**Table 2:** Behavioral changes in the adult zebrafish males exposed to different concentrations of ZnO-NPs for 96 h.

Behavior	ZnO-NPs Concentration (mg/L)								
	Control	0.05	0.1	0.2	0.4	0.8	1.6	3.2	6.4
Air gulping	−	−	−	−	−	+	+	++	++++
Respiratory distress	−	−	−	−	−	+	+	++	++++
Sluggish movement	−	−	−	−	−	+	+	++	++++
Uncoordinated swimming	−	−	−	−	+	+	++	+++	++++
Hyperventilation	−	−	−	−	−	+	++	++++	++++

None −, mild +, moderate ++, strong +++, very strong ++++.

**Table 3:** Effects of *D. salina* on the Zn residues accumulation (µg/gm wet weight) in the whole body of the treated male zebrafish (Means ± S.D.).

Groups	Zn Residues (µg/g Wet Weight)
Control	18.47 ± 0.25 ^d^
D1	13.21 ± 0.01 ^e^
D2	10.02 ± 0.01 ^f^
ZnO	60.85 ± 0.02 ^a^
ZnD1	41.23 ± 0.01 ^b^
ZnD2	30.09 ± 0.53 ^c^
SEM	4.30
* *p* value	< 0.001

Values are mean ± SE, values are not sharing a common superscript letter (a, b, c, d, e, f) differ significantly at *p* < 0.05. * *p*- overall treatment.

**Table 4:** Whole body composition (% wet weight basis).

Composition	Control	D1	D2	ZnO	ZnD1	ZnD2	* *p* Value
Moisture (%)	75.13 ± 0.01	76.17 ± 0.02	75.83 ± 0.01	76.03 ± 0.01	76.18 ± 0.01	76.61 ± 0.01	0.610
Ash (%)	4.86 ± 0.02	4.61 ± 0.01	4.62 ± 0.01	5.67 ± 0.02	5.01 ± 0.01	4.98 ± 0.08	0.104
Crude lipids (%)	6.21 ± 0.02 ^a^	5.40 ± 0.01 ^b^	5.02 ± 0.01 ^c^	3.07 ± 0.03 ^f^	4.05 ± 0.03 ^e^	4.73 ± 0.01 ^d^	<0.001
Crude protein (%)	13.91 ± 0.01	13.52 ± 0.02	13.40 ± 0.03	13.20 ± 0.01	13.44 ± 0.05	13.47 ± 0.56	0.100

Values are mean ± SE, values within each row are not sharing a common superscript letter (a, b, c, d, e, f) differ significantly at *p* < 0.05. * *p*- overall treatment.

## Data Availability

The data presented in this study are available on request from the corresponding authors.
